# Correction: Electrochemical behaviour and analysis of Zn and Zn–Ni alloy anti-corrosive coatings deposited from citrate baths

**DOI:** 10.1039/d0ra90137g

**Published:** 2020-12-22

**Authors:** Shams Anwar, Yahui Zhang, Faisal Khan

**Affiliations:** Centre for Risk, Integrity and Safety Engineering (C-RISE), Faculty of Engineering & Applied Science, Memorial University St. John’s NL A1B 3X5 Canada fikhan@mun.ca

## Abstract

Correction for ‘Electrochemical behaviour and analysis of Zn and Zn–Ni alloy anti-corrosive coatings deposited from citrate baths’ by Shams Anwar *et al.*, *RSC Adv.*, 2018, **8**, 28861–28873, DOI: 10.1039/C8RA04650F.

The authors regret a mistake in the author names of ref. 44. The correct reference is shown below as ref. 1.

The Royal Society of Chemistry apologises for these errors and any consequent inconvenience to authors and readers.

## Supplementary Material
